# Culture-negative cryptogenic splenic abscess in an immunocompetent host with recurrent misdiagnosis as pleural effusion: a case report with a review of the literature

**DOI:** 10.3389/fmed.2026.1874636

**Published:** 2026-06-26

**Authors:** Andrio Palayukan, Ummi Maimunah

**Affiliations:** 1 Department of Internal Medicine, Faculty of Medicine, Airlangga University, Surabaya, Indonesia; 2 Division of Gastroenterohepatology, Faculty of Medicine, Airlangga University, Surabaya, Indonesia

**Keywords:** abscess, delayed diagnosis, immunocompetence, pleural effusion, spleen diseases, splenectomy

## Abstract

Splenic abscess is a rare yet potentially life-threatening condition, often challenging to diagnose due to nonspecific clinical presentations. This case report describes a 52-year-old immunocompetent male who initially presented with fever and left-sided abdominal pain, and had been previously hospitalized and treated for pneumonia and left pleural effusion—a misattribution that delayed the definitive diagnosis—with transient clinical improvement only to return 2 weeks later with recurrent symptoms. Initial laboratory findings revealed leukocytosis, raised C-Reactive Protein (CRP) and elevated procalcitonin, while computed tomography (CT) imaging confirmed a splenic abscess. The patient underwent a combination of broad-spectrum antibiotic therapy and surgical intervention. Pus culture yielded no bacterial growth. Post-treatment, the patient’s condition markedly improved, with resolution of symptoms and successful hospital discharge. In immunocompetent patients with no identifiable predisposing condition, splenic abscess may hide behind the facade of recurrent pleural effusion—and culture negativity, far from a diagnostic dead end, should accelerate the decision for surgical intervention rather than delay it.

## Introduction

Splenic abscess is a rare but potentially life-threatening condition, with reported mortality of 7–14% despite contemporary antimicrobial and surgical advances (1, 2). Abscess formation may arise through hematogenous seeding from a distant infectious focus, direct extension from adjacent structures, or post-procedural complications (3, 4). Established predisposing conditions include infective endocarditis, abdominal trauma, intravenous drug use, haemoglobinopathies, and immunocompromising states including HIV/AIDS, uncontrolled diabetes mellitus, and immunosuppressive therapy (5). Diagnosis is frequently delayed owing to non-specific presentation, which may closely mimic pneumonia, pleuritis, or other intra-abdominal conditions (2). Contrast-enhanced CT remains the gold standard (6), while culture-negative results are not infrequent, attributable to prior antibiotic exposure or fastidious organisms (5).

A particularly underreported subset—cryptogenic splenic abscess in immunocompetent individuals without identifiable predisposing conditions—presents a compounded diagnostic challenge. We report a case of cryptogenic, culture-negative splenic abscess in an immunocompetent male previously treated for presumed pneumonia and pleural effusion, illustrating the diagnostic pitfalls and the critical role of timely surgical intervention.

### Case description

A 52-year-old immunocompetent male—with no history of diabetes mellitus, haematological malignancy, HIV infection, or immunosuppressive therapy—was referred to the emergency department with a one-month history of progressive left upper abdominal pain, intermittent fever, and slight dyspnea. Notably, 2 weeks prior, he had been hospitalized at another facility for 1 week and treated for community-acquired pneumonia and left pleural effusion (Supplementary Figure S1) with levofloxacin, with only transient improvement before symptomatic recurrence prompted the current referral.

On physical examination, pain was rated 6/10 (NRS), body temperature was 38.5 °C, conjunctival pallor was noted, breath sounds were diminished at the left lung base, and left hypochondriac tenderness with localized muscular guarding was present without peritoneal signs. Laboratory investigations revealed anaemia, leukocytosis, elevated ESR and CRP. Procalcitonin was within normal limits on admission but rose significantly at one-week re-evaluation (Table 1). Serological screening—Anti-HIV, HBsAg, and Anti-HCV—was unremarkable, excluding underlying immunosuppression. Fasting blood glucose was 103 mg/dL, below the diagnostic threshold for diabetes mellitus. Peripheral blood smear demonstrated no morphological abnormalities suggestive of haemoglobinopathy. Formal haemoglobin electrophoresis was not performed. Blood cultures yielded no bacterial growth. In view of the diminished breath sounds at the left lung base, thoracic ultrasonography was performed and demonstrated left-sided pleural effusion with an estimated volume of 408.48 mL (Supplementary Figure S2). Abdominal ultrasonography demonstrated a slightly hypoechoic splenic lesion measuring 6.04 × 4.14 cm with ill-defined borders (Supplementary Figure S2). In the context of persistent fever and neutrophil-predominant leukocytosis, these findings raised clinical suspicion for pyogenic splenic abscess as the primary working diagnosis. Empirical therapy with ceftriaxone 1 g every 12 h (targeting gram-negative enteric organisms) and metronidazole 500 mg every 8 h (providing coverage against anaerobes) was initiated.

**Table 1:** Laboratory of patient during admission.

Parameter	Timeline		
	On admission	1 week during admission	3 days after surgery
Haemoglobin (g/dl)	10.2	7.7	10.5
Leukocyte (/mm3)	24,590	22,160	13,400
Haematocrit (%)	32.3	23.8	33.5
Platelet (/mm3)	504,000	554,000	585,000
MCV (fl)	91.2	94.1	95.4
MCH (pg)	28.8	30.4	29.9
MCHC (g/dl)	31.6	32.4	31.3
Eosinophil (%)	0.2	0.7	4.3
Basophil (%)	0.2	0.2	0.5
Neutrophil (%)	88.7	82.2	74.3
Lymphocyte (%)	6.1	11.1	17
Monocyte (%)	4.8	5.8	3.9
Erythrocyte sedimentation rate (mm/h)	92	126	
SGOT (U/L)	7.0	103.0	7.7
SGPT (U/L)	7.6	97.8	4.9
BUN (mg/dL)		15.0	
Creatinine (mg/dL)		0.9	
Fasting Blood Glucose (mg/dL)	103		
Sodium (mmol/L)		131.0	
Potassium (mmol/L)		3.7	
Chloride (mmol/L)		99.0	
C-Reactive Protein (mg/L)	18		
Procalcitonin (ng/mL)	0.28	1.12	

Following 1 week of antibiotic therapy, re-evaluation demonstrated partial symptomatic improvement; however, leukocytosis and inflammatory markers persisted, and a decline in hemoglobin was observed (Table 1) raising concern for gastrointestinal blood loss as the likely source of acute haemoglobin decline. Gastrointestinal bleeding was excluded by a negative fecal occult blood test (FOBT), though no formal endoscopic was performed. No invasive or surgical procedures were performed during the admission period that could account for significant blood loss. Patient received 3 units of packed red cell transfusion. Repeat ultrasonography revealed interval progression to multiple hypoechoic splenic lesions. Contrast-enhanced CT confirmed two hypodense splenic lesions with irregular margins and peripheral rim enhancement—the largest measuring 6.7 × 2.6 × 4.6 cm—consistent with multilocular abscess formation (Figure 1) with no portal thrombosis reported. The multiplicity of lesions and failure of antibiotic therapy precluded percutaneous drainage and informed the decision to proceed with splenectomy. Echocardiography excluded infective endocarditis as a predisposing aetiology.

**Figure 1:**
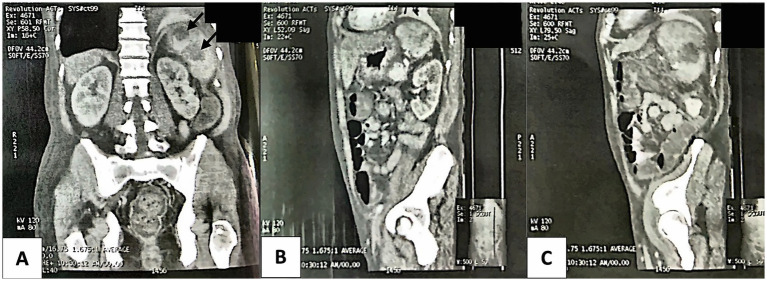
Contrast-enhanced computed tomography (CT) of the abdomen demonstrating multiple splenic abscesses. **(A)** Coronal reconstruction reveals multiple hypodense lesions within an enlarged splenic parenchyma with ill-defined borders and irregular margins (arrows), consistent with multilocular abscess formation. The heterogeneous enhancement pattern following contrast administration reflects the suppurative nature of the lesions. **(B,C)** Sagittal reconstructions further delineate the craniocaudal extent of the splenic lesions and their relationship to the left hemidiaphragm, providing additional characterisation of lesion distribution and morphology relevant to surgical planning. The multiplicity and multilocular configuration of the abscesses precluded percutaneous drainage as a viable therapeutic option.

Histopathological examination of the resected spleen (10 × 6.5 × 6.4 cm) revealed diffuse polymorphonuclear infiltration, lymphocytes, plasma cells, and histiocytes against a background of extensive necrotic debris, consistent with chronic suppurative inflammation (Figure 2). No granulomata, fungal elements, or malignant infiltration were identified, effectively excluding tuberculous, fungal, and neoplastic aetiologies.

**Figure 2:**
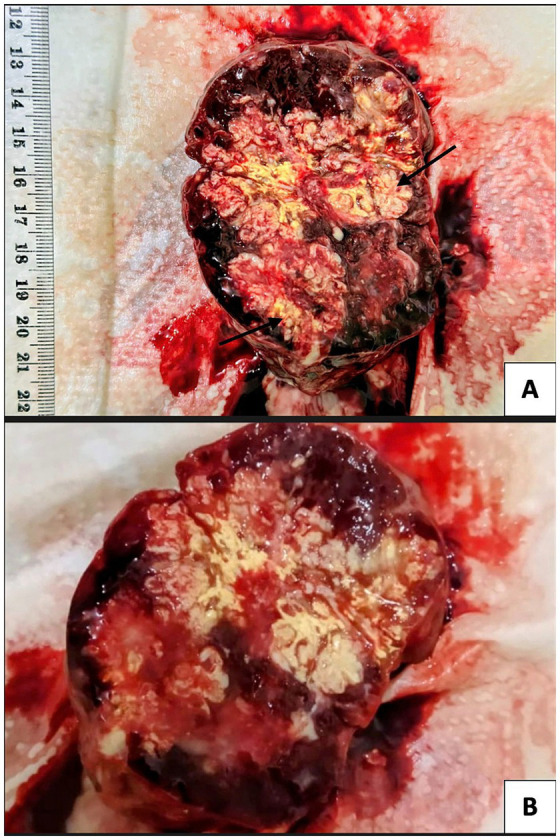
Gross pathological examination of the resected spleen following open splenectomy. **(A)** Cross-sectional view of the splenic specimen with ruler for scale, demonstrating multiple confluent yellowish-white areas of suppurative necrosis (arrows) distributed throughout the dark-red splenic parenchyma, consistent with multilocular abscess formation. The necrotic foci show irregular borders with surrounding haemorrhagic parenchyma, reflecting an advanced suppurative process. **(B)** Close-up view of the cut surface further delineating the extent and distribution of the necrotic material, with central creamy purulent exudate surrounded by congested and friable splenic tissue — macroscopic findings corroborated by subsequent histopathological confirmation of chronic suppurative inflammation.

Postoperatively, the patient was managed in the intensive care unit for 2 days. Antibiotic therapy was empirically escalated to cefoperazone-sulbactam 2 g every 12 h and metronidazole 500 mg every 8 h, given the failure of initial therapy and absence of culture guidance. Post-splenectomy vaccinations against *Streptococcus pneumoniae*, *Neisseria meningitidis*, and *Haemophilus influenzae* type b were administered prior to discharge. By post-operative day seven, the patient was afebrile, haemodynamically stable, tolerating oral intake, with normalised haemoglobin and leukocyte counts (Table 1). He was discharged with outpatient follow-up scheduled 7 days thereafter (see Figure 3).

**Figure 3:**
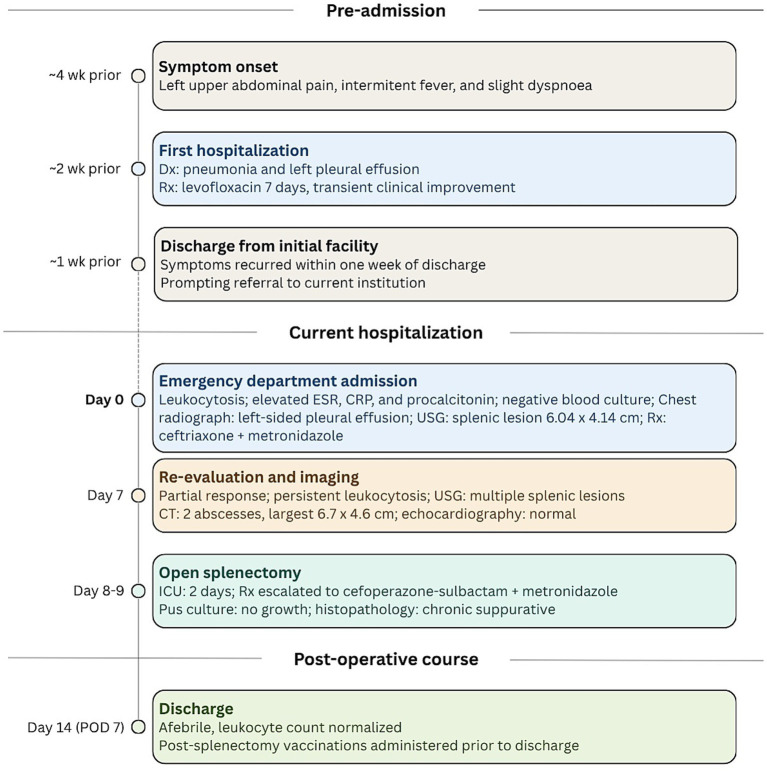
Clinical timeline of the patient’s episode of care. Key events are presented chronologically from initial symptom onset through outpatient follow-up. The dashed segment of the vertical axis between the pre-admission and current hospitalization phases indicates the approximate time interval between discharge from the initial facility and the current referral. Day 0 denotes the date of emergency department admission to the current institution.

## Discussion

The clinical course unfolded in two distinct phases: a phase of non-recognition, in which the underlying splenic abscess was unidentified at an outside facility and treatment was directed solely at the pulmonary manifestation without achieving source control; and a phase of recognized but medically refractory disease during the current admission, where prompt identification was followed by antibiotic failure necessitating splenectomy. These compounding phases account for the protracted clinical course and collectively illustrate both the diagnostic and therapeutic challenges inherent to this condition.

Splenic abscess is an uncommon but potentially lethal condition, carrying mortality rates of 7–14% despite contemporary antimicrobial and surgical advances (1, 2). Although immunosuppression, infective endocarditis, and haematological malignancy represent well-established predisposing conditions, a distinct and underreported subset—cryptogenic splenic abscess occurring in immunocompetent individuals without any identifiable risk factor—presents a unique diagnostic challenge. The absence of classical predisposing features may paradoxically reduce clinical suspicion at precisely the moment vigilance is most critical. The present case illustrates how an unrecognised splenic abscess may masquerade as recurrent pulmonary disease until the true diagnosis is eventually established.

Several differential diagnoses required systematic exclusion before the diagnosis of splenic abscess could be considered secure. Splenic cysts, whether congenital or post-traumatic, typically appear on CT as well-defined homogeneous lesions following fluid attenuation, without significant wall enhancement, which contrasts sharply with the irregular margins and peripheral rim enhancement observed in our patient (7). Splenic infarction was considered given the left-sided pain but excluded by the histopathological finding of neutrophil-rich suppurative inflammation rather than coagulative necrosis (8). Splenic lymphoma and metastatic disease were set aside following the absence of malignant cells or atypical lymphoid infiltrate on histopathology. Tuberculous splenic abscess warranted particular consideration given our endemic setting; however, the resected specimen showed no caseating granulomas, epithelioid macrophages, or Langhans-type giant cells characteristic of mycobacterial involvement (9). Fungal aetiology was excluded by negative GMS and PAS stains, which would have revealed hyphal or yeast forms had they been present (10). Amoebic splenic abscess remained a diagnosis that could not be formally dismissed and is addressed below.

The absence of any identifiable predisposing condition in our patient necessitated a systematic exclusion of occult aetiologies. Infective endocarditis was formally excluded by echocardiography; serological screening for HIV, HBsAg, and Anti-HCV was non-reactive; and no clinical features of immunodeficiency were identified. This workup places the case firmly within the cryptogenic category—a designation that is clinically meaningful, as the absence of expected risk factors may delay recognition. Evidence suggests that no identifiable source is found in a substantial proportion of splenic abscess cases, implying that cryptogenic presentations may be more prevalent than currently appreciated (4).

It bears emphasis that diabetes mellitus constitutes a well-recognized predisposing condition for pyogenic abscess at visceral sites, mediated through neutrophil dysfunction, impaired chemotaxis, and a chronic inflammatory microenvironment that facilitates bacterial seeding (4, 11). In the present case, the patient had no prior diagnosis of diabetes mellitus and admission fasting blood glucose was within normal limits (103 mg/dL), supporting the cryptogenic classification; nevertheless, clinicians should routinely screen for glycaemic dysregulation in all patients presenting with splenic abscess, as subclinical or newly diagnosed diabetes may represent an unrecognized predisposing condition.

The clinical presentation of splenic abscess is notoriously non-specific, accounting for much of the diagnostic delay encountered in practice. The classical triad of fever, left upper quadrant pain, and splenomegaly is present in only a minority of patients. Fever tends to be persistent and low-grade, often accompanied by night sweats and weight loss (12, 13). Left-sided abdominal pain—the most frequent presenting complaint—may be readily attributed to more common conditions affecting the left upper quadrant, including gastric pathology, musculoskeletal pain, or left-sided pleuropulmonary disease, compounding the diagnostic challenge. Particularly deceptive is the thoracic manifestation: left-sided pleural effusion arising as a sympathetic response to subdiaphragmatic inflammation may be misattributed to primary pulmonary pathology, resulting in an erroneous diagnosis of pneumonia or pleuritis (14). This pitfall carries significant consequences in terms of delayed source control. Additional systemic features—anorexia, nausea, and fatigue—further obscure the clinical picture (4, 15). An alternative interpretation warrants consideration: rather than representing solely a misdiagnosis, the initial pulmonary infection may itself have served as the primary source of haematogenous seeding to the spleen (16). Community-acquired pneumonia caused by encapsulated organisms, most notably *Streptococcus pneumoniae* and *Klebsiella pneumoniae*, is a recognised albeit uncommon precipitant of splenic abscess through bacteraemic spread (17). Under this interpretation, the left pleural effusion may reflect both a pulmonary infectious process and a concurrent subdiaphragmatic sympathetic response—two pathological processes occurring in parallel rather than sequentially. The sterile cultures in this case preclude definitive determination of the aetiological sequence (4).

Our patient’s clinical course illustrates this pitfall with particular clarity. The temporal escalation of pain over 1 month is consistent with progressive abscess expansion. In retrospect, the pleural effusion most likely represented a sympathetic response secondary to subdiaphragmatic inflammation—a well-described but frequently overlooked phenomenon in which left-sided pleural fluid accumulates without direct pleural infection (14). The anatomical proximity of the spleen to the left hemidiaphragm facilitates transdiaphragmatic inflammatory signalling, producing pleural exudate that can mimic pneumonia (18, 19). Treating the pulmonary manifestation without addressing the splenic source predictably produced only transient benefit. Recurrent or refractory left pleural effusion unresponsive to pneumonia therapy should therefore prompt active investigation of subdiaphragmatic pathology.

Physical examination has well-recognized limitations in this condition. Splenic tenderness may be elicited on palpation, but splenomegaly is often absent—particularly in patients with early-stage disease—and percussion lacks specificity (14, 20). In our patient, examination revealed fever, conjunctival pallor, reduced breath sounds at the left lung base, and localized tenderness with muscular guarding in the left hypochondriac region—findings consistent with systemic infection but non-diagnostic in isolation. The absence of palpable splenomegaly is consistent with published series in which splenic enlargement is detectable in fewer than half of confirmed cases (20), reinforcing that a normal splenic examination does not exclude abscess formation.

Laboratory investigation provides indispensable but non-specific support. Leukocytosis with neutrophil predominance, elevated ESR, and raised CRP are the most consistently observed abnormalities (6). Liver and renal function tests, though non-specific, may reveal systemic involvement in sepsis-complicated cases (13, 21), though these markers are insufficient for definitive diagnosis and can be seen across a broad spectrum of infectious conditions (21). In our patient, admission findings included normocytic anaemia, neutrophil-predominant leukocytosis, elevated ESR, and raised procalcitonin—the latter suggesting systemic rather than localized infection.

The acute haemoglobin decline from 10.2 to 7.7 g/dL within 1 week is inconsistent with anemia of chronic inflammation, which develops gradually over weeks to months through hepcidin-mediated functional iron deficiency (22, 23). Rather, this acute decline raises concern for gastrointestinal blood loss, though formal endoscopic workup was not performed during this admission—appropriately investigated by FOBT in this case—or hemorrhagic contribution from the suppurative process itself. The subsequent haemoglobin recovery to 10.5 g/dL following 3 units packed red cell transfusion supports the hemorrhagic basis of this acute decline.

A clinically significant hepatic transaminase rise—SGOT from 7 to 103 U/L and SGPT from 7.6 to 97.8 U/L—warranted differential consideration of sepsis-induced hepatic dysfunction, drug-induced liver injury from antibiotic therapy, or haematogenous hepatic seeding (13, 24, 25). The absence of jaundice, preserved synthetic function, and transaminase normalization following splenectomy favours sepsis-induced hepatic stress as the most probable mechanism. Hepatic involvement should nonetheless be systematically monitored in all patients with sepsis-complicated splenic abscess.

Blood cultures yielded no bacterial growth—a finding not uncommon in splenic abscess and attributable to prior antibiotic exposure, fastidious or slow-growing organisms poorly recovered on standard aerobic media, and reduced viable organism burden within the chronic, walled-off abscess at the time of drainage (4). The inability to identify a causative organism necessitated continued empirical broad-spectrum coverage postoperatively. Pus specimens should routinely be submitted for aerobic, anaerobic, fungal, and—in endemic regions—mycobacterial cultures simultaneously, as each additional medium improves the likelihood of microbiological confirmation.

An important differential diagnosis that warrants explicit consideration in this case is amoebic splenic abscess. *Entamoeba histolytica* is a well-recognized cause of extraintestinal abscess formation—most commonly hepatic but occasionally splenic—particularly in tropical and subtropical endemic regions including Indonesia (26). Importantly, amoebic abscesses characteristically produce sterile cultures on standard bacteriological media, rendering culture negativity diagnostically inconclusive in this context (26, 27). In our patient, amoebic serology and PCR analysis of the pus specimen were not performed—a recognized limitation of this case. The histopathological findings of chronic suppurative inflammation without trophozoites or characteristic tissue invasion patterns do not support amoebic aetiology, though histopathology alone cannot formally exclude it. In endemic settings, serological testing for *E. histolytica* and PCR-based analysis of pus specimens should be incorporated into the routine microbiological workup of culture-negative splenic abscess.

Regarding imaging, ultrasonography is the most accessible first-line modality, typically demonstrating hypoechoic lesions with ill-defined margins (28), though its accuracy is operator-dependent and limited by bowel gas interference (29). Contrast-enhanced CT remains the gold standard, providing superior lesion characterization and assessment for complications such as rupture or perisplenic extension (30). MRI may assist in equivocal cases, particularly to distinguish abscess from cystic or necrotic tumour (6). In our patient, serial imaging revealed a solitary hypoechoic lesion on initial ultrasonography that progressed to multiple lesions 1 week later—a radiological evolution that fundamentally altered the management strategy and underscores that interval progression in the absence of clinical improvement should be regarded as a decisive signal to escalate diagnostic and therapeutic strategies.

Management is guided by lesion characteristics, clinical trajectory, and the patient’s condition. Broad-spectrum antibiotic therapy forms the backbone of initial treatment (1, 13), while percutaneous drainage under imaging guidance offers a minimally invasive option for unilocular or anatomically accessible lesions that preserves splenic function (1, 31). Splenectomy is reserved for cases where conservative measures fail or complications have developed (2). In our patient, 7 days of ceftriaxone and metronidazole produced only partial clinical response—with subjective improvement in fever and pain—yet failed to produce meaningful laboratory or radiological improvement. The multilocular nature of the abscesses, consistently associated with higher percutaneous drainage failure rates, made conservative continuation insufficient, and splenectomy was undertaken. By post-operative day seven, clinical status and laboratory parameters had normalized, permitting safe discharge.

The antibiotic course illustrates a key principle in culture-negative splenic abscess management: empirical escalation guided by clinical and radiological trajectory rather than microbiological data. Given the failure of ceftriaxone-based therapy and the absence of culture guidance, escalation to cefoperazone-sulbactam was undertaken on empirical grounds. Cefoperazone-sulbactam offers broader coverage against beta-lactamase-producing Enterobacteriaceae and non-fermenting gram-negative bacilli that may evade third-generation cephalosporins and represent plausible causative agents in cryptogenic cases (1). Successful clinical and biochemical recovery without ever identifying a causative organism affirms that in culture-negative cases, escalation decisions must be driven by the overall clinical trajectory rather than deferred awaiting microbiological confirmation that may never arrive.

Finally, post-splenectomy care demands attention to the long-term consequences of functional asplenia. Splenectomised patients face lifelong risk of overwhelming post-splenectomy infection—a rapidly progressive, potentially fatal septicaemia caused predominantly by encapsulated organisms including *Streptococcus pneumoniae*, *Neisseria meningitidis*, and *Haemophilus influenzae* type b. Current guidelines universally recommend vaccination against these organisms prior to or within 2 weeks of splenectomy, alongside patient education regarding prompt presentation for febrile illness (32, 33). In our patient, post-splenectomy vaccination was administered prior to discharge.

Several limitations warrant acknowledgement. First, the full scope of microbiological investigation—including anaerobic, fungal, and mycobacterial cultures—could not be confirmed retrospectively; the possibility of a causative organism evading standard aerobic culture conditions cannot be entirely excluded. Second, comprehensive immunological evaluation, including complement levels and immunoglobulin quantification, was not performed, and an occult primary immunodeficiency contributing to the patient’s susceptibility cannot be formally ruled out. Formal haemoglobinopathy screening via haemoglobin electrophoresis was not performed, and a subclinical haemoglobinopathy cannot be entirely excluded as a contributing factor. Amoebic serology and PCR analysis of pus for *Entamoeba histolytica* were not performed, and amoebic aetiology cannot be formally excluded on the basis of histopathology alone. Additionally, a dedicated Doppler ultrasound examination of the portal vein was not formally documented during hospitalization. Given the presence of hepatic enzyme elevation in this patient, we recommend that future cases presenting with splenic abscess and concurrent hepatic enzyme abnormalities undergo dedicated portal vein Doppler evaluation to formally rule out thrombotic complications. Finally, as a single case report from one institutional context, the generalizability of these findings is inherently limited and the conclusions should be interpreted as hypothesis-generating rather than practice-defining.

## Conclusion

Despite its rarity, splenic abscess should be actively considered in the differential diagnosis of any patient presenting with left upper quadrant pain accompanied by an infectious syndrome—defined by fever, leukocytosis, and elevated inflammatory markers—particularly in the presence of predisposing conditions such as diabetes mellitus, haematological disease, or immunosuppression. This case documents a cryptogenic, culture-negative splenic abscess in an immunocompetent male without identifiable predisposing conditions. Recurrent left-sided pleural effusion refractory to standard pneumonia therapy should prompt active investigation of subdiaphragmatic pathology, as splenic abscess may present exclusively through sympathetic thoracic manifestations. From a therapeutic standpoint, culture negativity is neither a diagnostic dead end nor a barrier to cure; timely surgical source control, guided by clinical and radiological trajectory, remains life-saving. Clinicians encountering unexplained recurrent left pleural effusion with persisting inflammatory markers should maintain a low threshold for cross-sectional abdominal imaging—the single most consequential determinant of outcome.

## Data Availability

The original contributions presented in the study are included in the article/Supplementary material, further inquiries can be directed to the corresponding author.

## Patient perspective

The patient reported experiencing symptoms for approximately 1 month before a definitive diagnosis was established, describing the period of misdiagnosis as both physically and emotionally distressing. Upon learning that splenectomy was necessary, he expressed initial apprehension, but stated that clear communication from the clinical team helped him understand and accept the decision. He reported rapid recovery following surgery and found the pre-discharge education regarding post-splenectomy precautions reassuring. The patient consented to publication and expressed hope that his experience might facilitate earlier diagnosis in similar cases.
