# Supplementary material for: Culture-negative cryptogenic splenic abscess in an immunocompetent host with recurrent misdiagnosis as pleural effusion: a case report with a review of the literature
Source: Front Med (Lausanne). 2026 Jun 26;13:1874636. doi: 10.3389/fmed.2026.1874636 (PMC13349773; doi:10.3389/fmed.2026.1874636)
Supplement: Supplementary file 1 [file Table_1.DOCX]

**
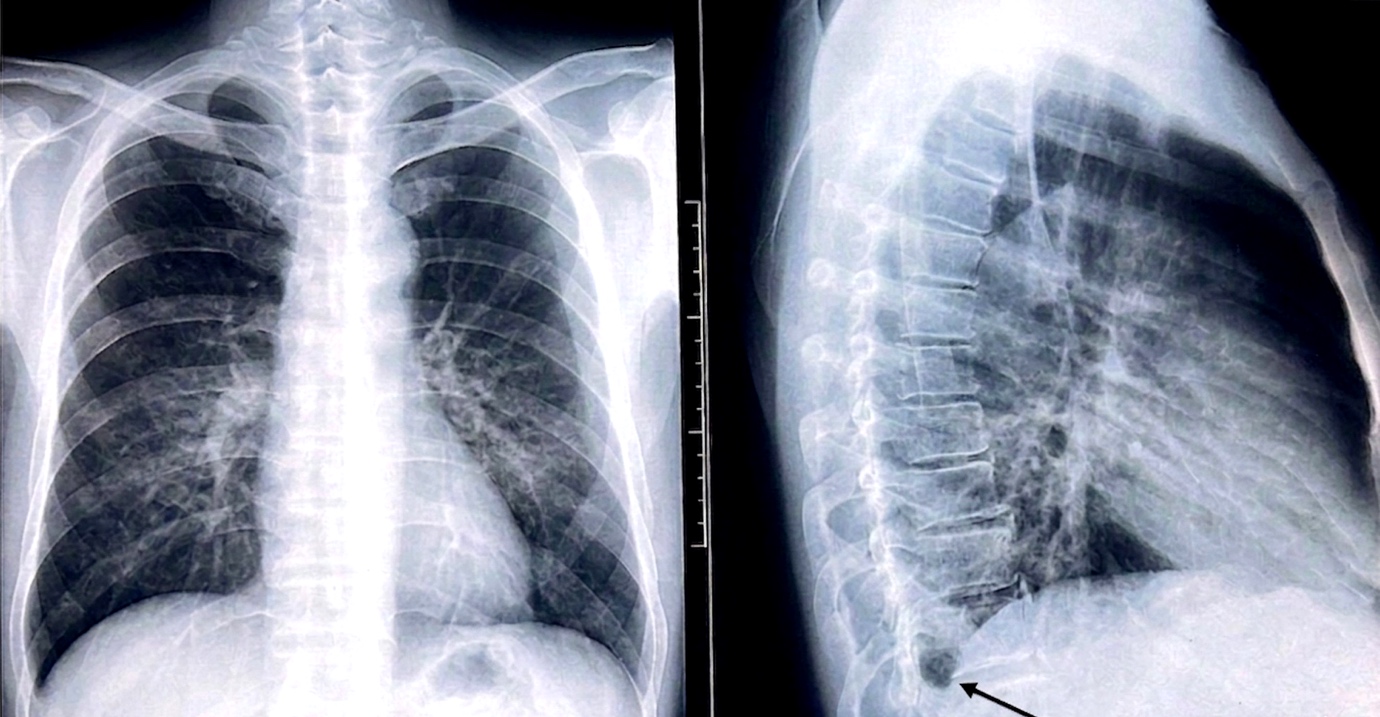
**

B

A

**Supplementary Figure S1.** Chest radiography performed at the initial facility during the index hospitalization, one month prior to current admission. (A) Posteroanterior projection demonstrating significant left lower zone opacification with obliteration of the left hemidiaphragm silhouette and blunting of the left costophrenic angle. (B) Lateral projection demonstrating posterior basal opacification consistent with layering pleural fluid (arrow, added by authors). These findings were attributed to community-acquired pneumonia and left pleural effusion.

**
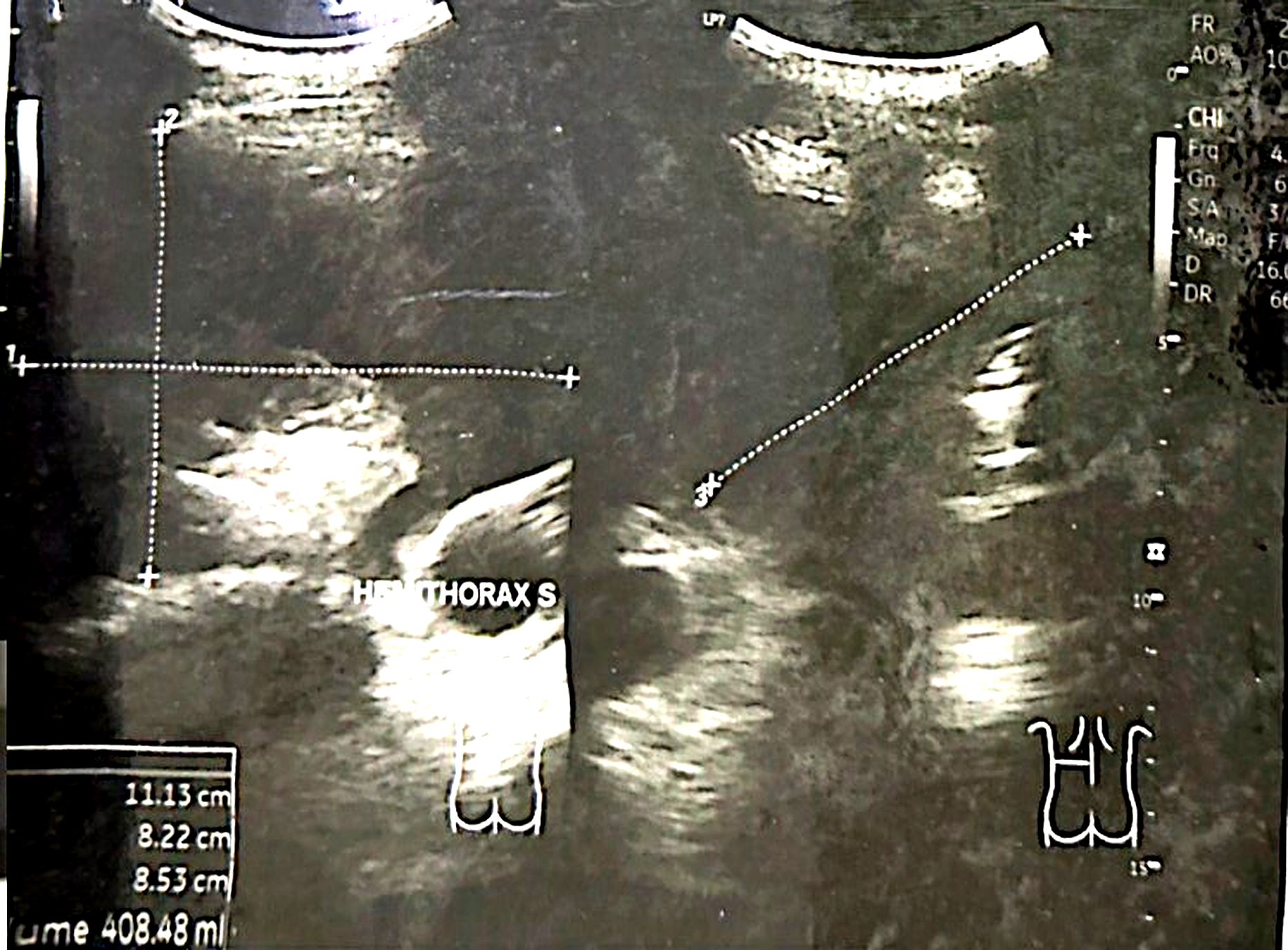
**

**Supplementary Figure S2**. Thoracic ultrasonography of the left hemithorax demonstrating a significant pleural effusion. Multiple imaging planes reveal an anechoic fluid collection within the left pleural space, with measured dimensions of 11.13 × 8.53 × 8.22 cm and an estimated volume of 408.48 mL. These findings are consistent with sympathetic pleural effusion secondary to subdiaphragmatic inflammation, corroborating the diminished breath sounds elicited on auscultation and the thoracic manifestation documented at the initial facility.

**
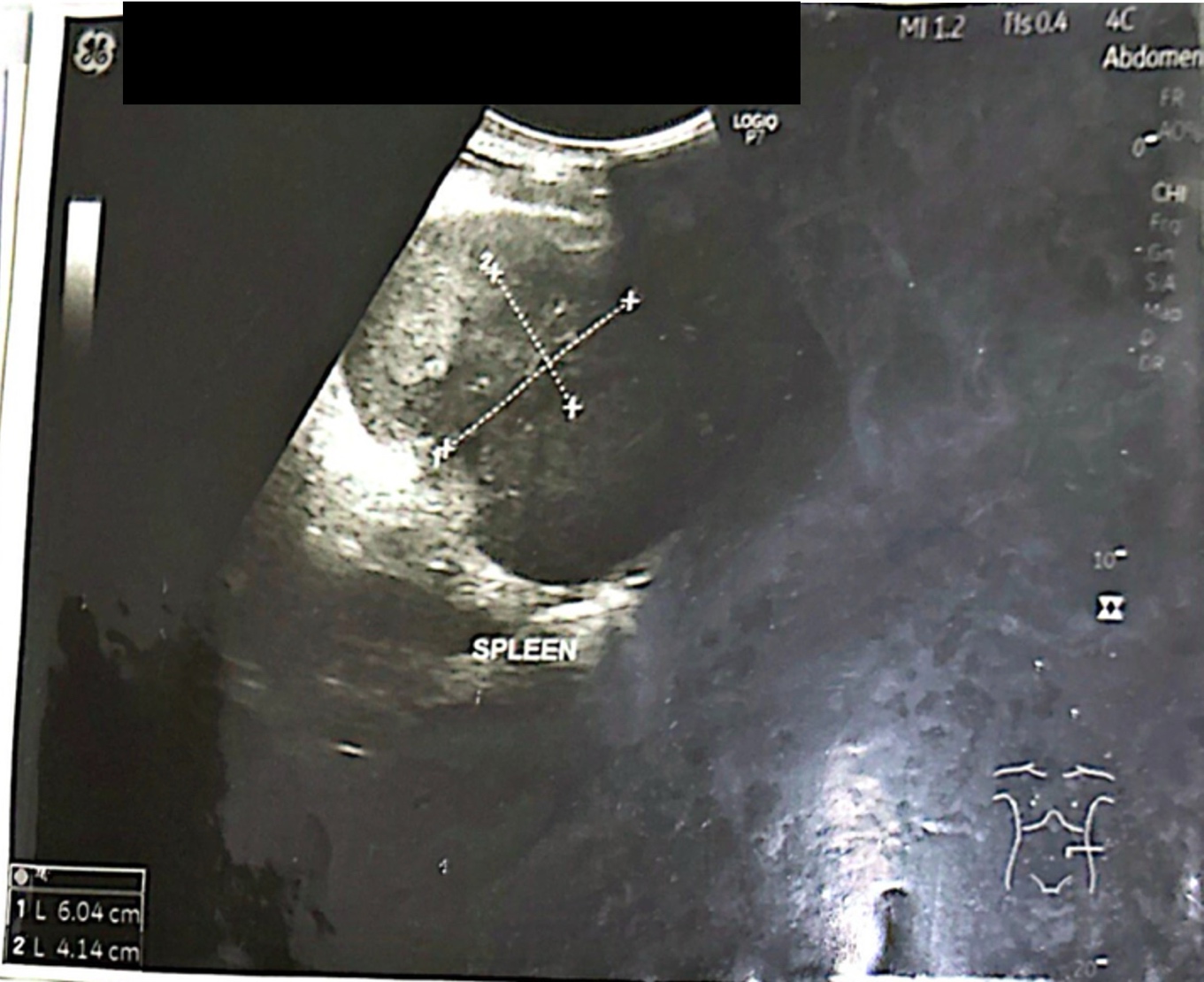
**

**Supplementary Figure S3.** Abdominal ultrasonography on admission demonstrates a splenic lesion with heterogeneous and slightly hypoechoic internal architecture measuring 6.04 × 4.14 cm (indicated by electronic callipers), with ill-defined borders and irregular margins within the splenic parenchyma. The mixed echogenicity pattern is consistent with an evolving suppurative infectious process. At this stage, the lesion appeared solitary, without evidence of additional lesions; however, interval progression to multiple hypoechoic splenic lesions was demonstrated on follow-up ultrasonography one week later.

**
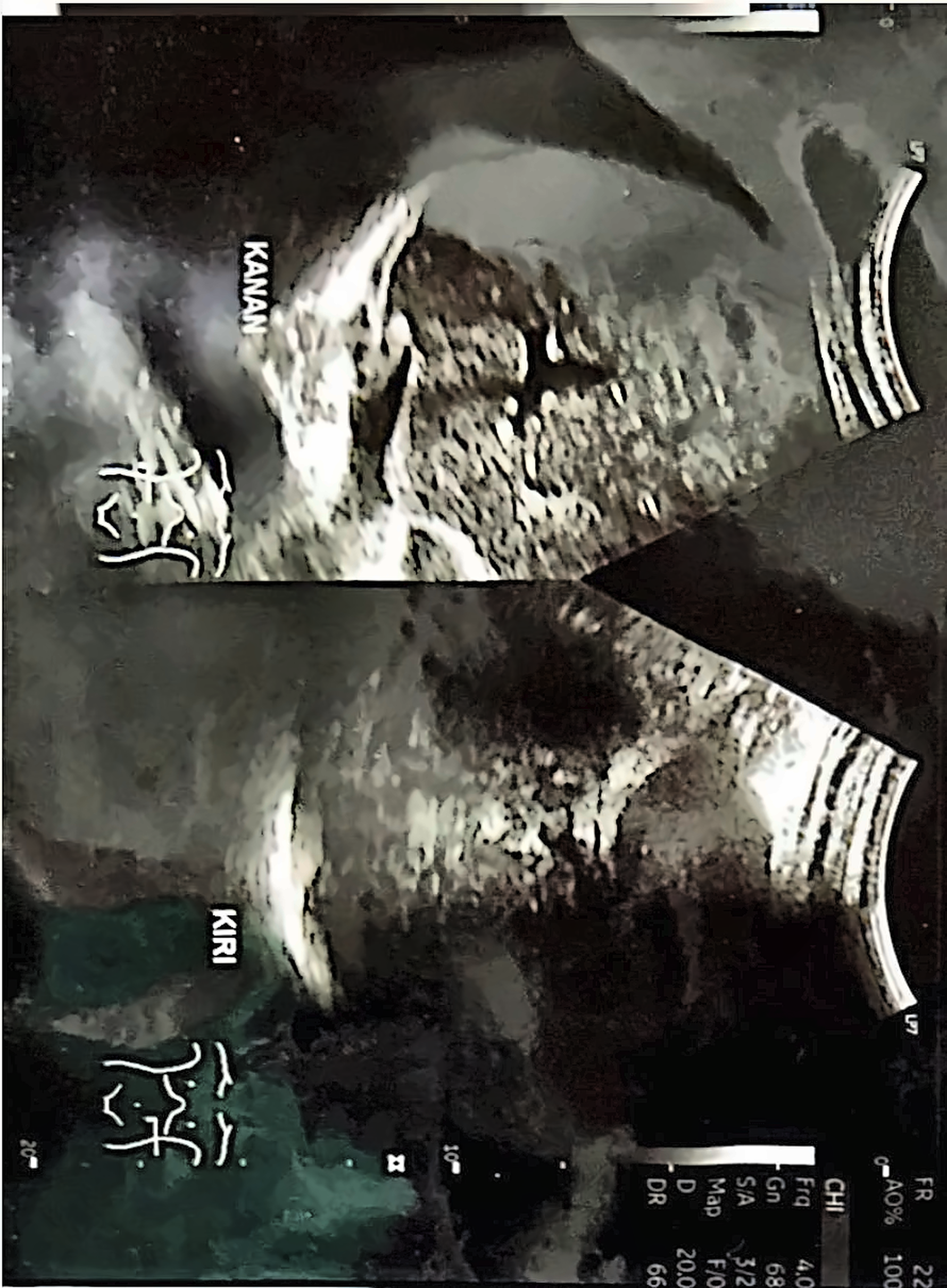
**

**Supplementary Figure S4. Abdominal ultrasound of the liver.** Right lobe and left lobe views demonstrating normal liver size with homogeneous parenchymal echotexture, smooth surface, and sharp margins. No focal lesions, intrahepatic biliary dilatation, or vascular abnormalities were identified.
